# The role of conjunctival biopsy in the diagnosis of granulomatosis with polyangiitis

**DOI:** 10.1186/s12348-014-0033-9

**Published:** 2015-01-23

**Authors:** Roxana Ursea, Dawn De Castro, Trent J Bowen, Chi-Chao Chan

**Affiliations:** Department of Ophthalmology and Vision Science, College of Medicine, University of Arizona, Tucson, AZ 85711 USA; Oculoplastics Service, Massachusetts Eye and Ear Infirmary, Boston, MA 02114 USA; College of Medicine, University of Arizona, Tucson, AZ 85711 USA; Immunopathology Section, National Eye Institute, Bethesda, MD 20892 USA

**Keywords:** Conjunctivitis, Cornea, Ulceration, Biopsy, Granulomatosis with polyangiitis

## Abstract

**Background:**

The purpose of this study is to describe a patient who was diagnosed with granulomatosis with polyangiitis based on conjunctival biopsy. This study is a case report and review of the literature.

**Findings:**

A 48-year-old Caucasian woman presented with a 2-week history of a left eye peripheral corneal ulcer with adjacent conjunctivitis and a 4-month history of a non-resolving productive cough. Given her elevated serum perinuclear antineutrophil cytoplasmic antibody (P-ANCA) and erythrocyte sedimentation rate (ESR) levels as well as a chest computed topography (CT) that showed bilateral patchy infiltrates, suspicion of limited granulomatosis with polyangiitis with lung and ocular involvement was high. Because bronchoalveolar lavage was nondiagnostic for granulomatous disease, conjunctival biopsy was initially attempted in order to avoid a more invasive lung biopsy. The conjunctival biopsy revealed mixed subacute inflammatory mediators and vasculitis consistent with granulomatosis with polyangiitis.

**Conclusions:**

Conjunctival biopsy may be a valuable, minimally invasive method for diagnosing systemic granulomatosis with polyangiitis.

## Findings

### Background

Granulomatosis with polyangiitis is a rare, potentially life-threatening primary systemic vasculitis of unknown etiology. It was first described in 1931 by Heintz Klinger, a medical student, as a form of polyarteritis nodosa [1], but the clinical features were more fully delineated in 1937 by Frederick Wegener and the disease was also known as Wegener's granulomatosis [2]. The prevalence of granulomatosis with polyangiitis in the United States has been estimated at nearly 3 cases per 100,000 [3]. It is a multisystem inflammatory disease characterized in its ‘complete’ form by necrotizing granulomatous inflammation of the upper and lower respiratory tracts, systemic vasculitis predominantly affecting small vessels, and focal glomerulonephritis [4-6]. Patients without renal involvement have the ‘limited’ form of the disease [4,7-10].

Ocular manifestations are thought to be relatively common and occur in approximately 50% (range: 28% to 87%) of cases of generalized granulomatosis with polyangiitis [11,12]. Furthermore, ophthalmic disease may be the presenting symptom in 8% to 16% of patients [12,13]. Almost any ocular or adnexal tissue can be affected. According to Harman and Margo [14], the most common findings are proptosis, described in nearly one fifth of patients, as well as keratoscleritis. Proptosis can result either from primary orbital involvement or secondarily from extensive sinusitis. Other common ocular manifestations of granulomatosis with polyangiitis include conjunctivitis (4% to 15%) [4,13], episcleritis, scleritis, retinal and optic nerve vasculitis, nasolacrimal duct obstruction, uveitis, and dacryocystitis.

The diagnostic workup of granulomatosis with polyangiitis includes laboratory tests, imaging studies, and biopsy of affected tissues. Testing for serum antineutrophil cytoplasmic antibodies (ANCAs) has significant value in the diagnosis of Wegener's as well as other systemic vasculitides. Although cytoplasmic antineutrophil cytoplasmic antibody (C-ANCA) testing in Wegener's has a sensitivity of 85%, perinuclear antineutrophil cytoplasmic antibody (P-ANCA) testing has a much lower sensitivity of 10% [15].

While biopsy is an integral component of the diagnostic workup, the classic pathologic triad of parenchymal necrosis, vasculitis, and granulomatous inflammation is not commonly seen in extrapulmonary sites, with 91% of open lung biopsies yielding positive results [4] compared to 54% of orbital biopsies [16]. Only a few case reports have been published in the literature on the role of conjunctival biopsy in the diagnosis of systemic granulomatosis with polyangiitis [17-20]. Furthermore, to date, there have been no publications regarding the diagnosis of Wegener's based on conjunctival biopsy in the setting of elevated P-ANCA titers.

### Methods and results

#### Case report

A 48-year-old Caucasian woman presented with a 2-week history of left eye redness and pain. She had initially been treated by a local emergency room physician with ciprofloxacin 0.3% ophthalmic solution twice daily, which after 4 days did not alleviate her symptoms. Her primary care physician subsequently diagnosed her with allergic conjunctivitis and instructed her to stop the antibiotic drops and start topical allergy medications. When her symptoms worsened, the patient was placed on erythromycin ophthalmic ointment three times daily and referred to a cornea specialist in the community. This specialist diagnosed her with a left eye peripheral corneal ulcer and immediately referred her to the authors for further evaluation and treatment.

This patient's past medical history was significant for symptomatic cholelithiasis, mild hypertension, and a 4-month history of recurrent left lower lobe pneumonia for which her primary care physician had been treating her with multiple courses of cephalexin 500 mg orally three times daily as well as oral montelukast, a fluticasone inhaler, and an albuterol inhaler. Despite mild improvement in response to this regimen, her symptoms persisted. Computed topography (CT) imaging of the thorax with contrast was consequently performed, which was significant for left lower lobe lateral and posteromedial basilar segment patchy infiltrates that were suggestive of primary granulomatous disease, and she was referred to a pulmonologist. Bronchoalveolar lavage was negative for acid-fast bacilli and fungi and was nondiagnostic with regard to granulomatous disease.

**Figure 1:**
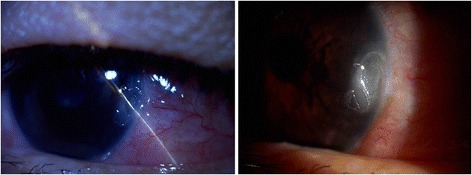
**External and slit lamp photos of the left eye showing corneal ulceration.**

**Figure 2:**
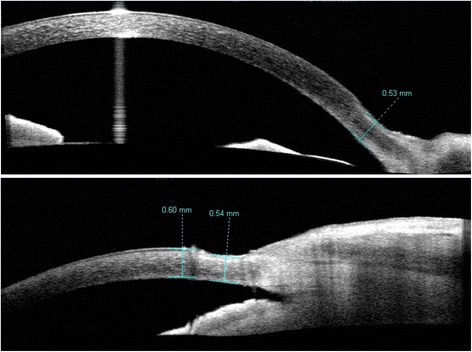
**Anterior segment optical coherence tomography (Visante) of the affected left cornea showing significant peripheral thinning.** Top: anterior segment scan; bottom: angle view.

On presentation to the authors, the patient reported redness and itchiness of the left eye with severe photophobia but no discharge. Her uncorrected vision was 20/20 right eye and 20/25 left eye. Her pupils were equally round and reactive to light with no afferent pupillary defect. Intraocular pressure measured 14 mmHg right eye and 11 mmHg left eye. Slit lamp examination of the right eye was within normal limits, and that of the left eye revealed 2+ left upper lid edema and a 2.2 × 0.8 mm corneal ulcer with rolled edges located at the limbus at 3 o'clock. There was 1+ diffuse conjunctival injection with 3+ chemosis and prominent tortuous vessels adjacent to the corneal ulcer (Figure 1). There was also a mild anterior chamber reaction with 1+ cells and no flare. The rest of the ocular examination, including dilated funduscopic exam, was within normal limits for both eyes. Anterior segment optical coherence topography (AS-OCT) was performed, which was significant for corneal thinning in the area of the ulcer, with the thinnest site measuring 560 μm (Figure 2), as well as thickening of the adjacent conjunctiva with cystic spaces and few central keratic precipitates.

Cultures were taken from the left eye corneal ulcer. Blood tests revealed a normal white blood cell count with a slightly elevated (6%) eosinophilic component, a normal hemoglobin level, and a normal platelet count. Erythrocyte sedimentation rate (ESR) was mildly elevated at 25. P-ANCA level (myeloperoxidase, MPO) was elevated at 21 (positive if ≥1.0). C-ANCA (proteinase 3, PR3) was negative. Rheumatoid factor level was elevated at 326, while anti-dsDNA antibodies and antinuclear antibodies (ANA) were negative. No casts or hematuria was noted on urine sedimentation analysis.

The patient was immediately started on gatifloxacin 0.3% ophthalmic solution four times daily in the left eye. Fortified vancomycin and tobramycin were later added, which were initially used every hour and gradually tapered to four times daily.

Minimal improvement was noted on clinical exam after 4 days of intensive topical antibiotic therapy, and corneal cultures were negative for bacterial, fungal, and viral organisms. There was a high suspicion of granulomatosis with polyangiitis given her elevated P-ANCA and ESR levels, as well as the patient's persistent pulmonary symptoms and chest CT findings suggestive of granulomatous disease. As above, her bronchoalveolar lavage was nondiagnostic, and therefore, a more definitive diagnostic approach was sought.

**Figure 3:**
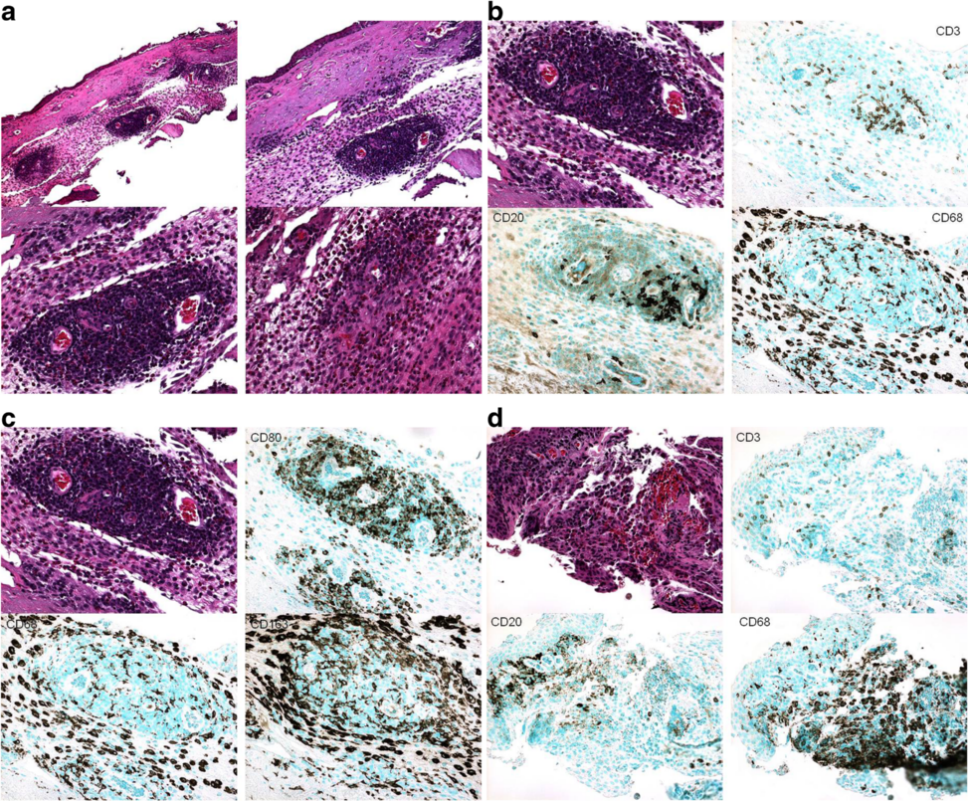
**Histopathology and immunopathology of the conjunctival biopsy. (a)** Severe conjunctival inflammation with prominent vasculitis in the deep substantia propria (hematoxylin & eosin, original magnifications: upper left ×100, upper right ×200, lower ×400). **(b-d)** Strong immunoreactivities to T cells (CD3), B cells (CD20), macrophages (CD68), M2 macrophages (CD163), and costimulatory signal (CD80) (avidin-biotin complex immunohistochemistry, original magnification: ×400).

Although open lung biopsy was considered in this patient's case, it was suggested that conjunctival biopsy first be attempted as a less invasive procedure. This biopsy was performed, and results were significant for diffuse infiltration of polymorphonuclear leukocytes (PMNs), eosinophils, lymphocytes, and macrophages in the substantia propria (Figure 3a). Many T- and B-lymphocytes, CD80+ cells (activated B cells and monocytes), and M2 (CD163+) macrophages were surrounding dilated blood vessels (Figure 3b,c). The diagnosis of the conjunctival biopsy showed severe subacute inflammation with aggregates of CD68+ macrophages (granulomatous formation) and vasculitis (Figure 3d), findings consistent with granulomatosis with polyangiitis.

These histopathological and immunopathological findings, together with the clinical signs and symptoms, treatment, and long-term prognosis of this rare disease, were discussed with the patient. The topical antibiotic drops were stopped, and the patient was referred to her primary physician for long-term systemic management. The patient had no recurrence of keratitis or uveitis at 1 and 3 months follow-up in the ophthalmology clinic.

### Discussions

The diagnosis of granulomatosis with polyangiitis or Wegener's is dependent not only solely on biopsy but also on the appropriate clinical presentation in conjunction with laboratory tests and imaging studies, namely chest radiography to rule out infiltrates or nodules. With regard to laboratory investigation, serum ANCA testing for granulomatosis with polyangiitis had become an essential component of the workup since the 1980s [21]. When neutrophils are fixed in alcohol, two different immunofluorescent patterns emerge - a diffuse granular fluorescence of the cytoplasm (i.e., C-ANCA) or a predominantly perinuclear pattern of staining (i.e., P-ANCA). The C-ANCA pattern of staining is most often directed against proteinase-3, and the P-ANCA pattern against myeloperoxidase. The sensitivity of C-ANCA has been reported to be as high as 85% to 96% in patients with complete Wegener's, but drops to 80% when including patients with limited disease. The P-ANCA pattern is considered an insensitive marker of Wegener's because it is only positive in 10% of patients with the disease [15]. Our patient demonstrated an elevated P-ANCA titer with a negative C-ANCA test and is therefore considered a relatively atypical case of granulomatosis with polyangiitis.

Ophthalmic involvement in granulomatosis with polyangiitis is an important manifestation, and histological diagnosis is essential for the disease [22]. Conjunctivitis is common and may be ulcerative, necrotic, and/or cicatricial [23]. In a recent review of 1,286 patients with systemic necrotizing vasculitides, 214 (16.6%) had ocular involvement; conjunctivitis was the most common documented finding of ocular presentation [24]. In this cohort, granulomatosis with polyangiitis was the most common disease that manifests with ophthalmic involvement.

With regard to cases that were diagnosed based on biopsy of the conjunctiva in particular, there are only a few case reports in the literature. Karakousis et al. [17] described an 18-year-old boy with scleritis, refractory sinusitis, and an elevated C-ANCA titer. He was diagnosed with granulomatosis with polyangiitis after his conjunctival biopsy revealed perivascular inflammation with areas of necrosis. In addition, Toh et al. [18] reported a 61-year-old woman who presented with mild proptosis, bulbar conjunctival ulceration adjacent to the limbus, and central retinal artery occlusion, along with marked nasal turbinate hypertrophy. Biopsy obtained during her turbinectomy demonstrated non-specific acute inflammation only, but when the margins of the conjunctival ulcer were biopsied, the histopathology showed pauci-immune granulomatous inflammation consistent with Wegener's. In another report [19], conjunctival biopsy in two patients with palpebral conjunctivitis revealed granulomatous infiltration with focal vasculitis in one patient and mixed inflammatory cellular reaction in the other. Finally, a 70-year-old man with cicatricial palpebral conjunctival inflammation and secondary trichiasis and marginal ectropion underwent palpebral conjunctival biopsy in order to rule out malignancy. The biopsy was significant for chronic granulomatous inflammation with multinucleated giant cells and areas of perivascular inflammation consistent with Wegener's [20]. In general, given the possible risk of surgically induced scleritis in patients with serological and/or clinical evidence of collagen vascular disease, performing a biopsy should be a well-thought-after decision. In our patient's case, given her nondiagnostic CT and BAL, a definitive diagnosis was imperative; the biopsy performed was able to elicit a conclusive diagnosis showing granulomatous inflammation in the conjunctiva. The immunopathological findings of CD80+ cells and M2 macrophages surrounding conjunctival vessels are novel and suggest activated B cells and monocytes that provide B7-1, a costimulatory signal protein needed for autoreactive T cell activation. The data support the important role for activated T cells in granulomatosis with polyangiitis [25,26].

### Conclusions

Conjunctival biopsy is a relatively simple, minimally invasive means of supporting the diagnosis of granulomatosis with polyangiitis. It can be considered as a possibility in patients who present with conjunctivitis and in which biopsy of other involved organs such as the nasal passages, lungs, or kidneys is contraindicated. Results of this type of biopsy will likely not include the full classic histopathological triad but, when considered in combination with the clinical presentation and other diagnostic findings, can be strongly supportive of the diagnosis. Larger studies are needed in order to more fully elucidate the diagnostic utility of conjunctival biopsy in patients with conjunctival involvement and suspected granulomatosis with polyangiitis.

## Consent

The patient presented in this case report agreed and gave her consent for the report to be published.
